# Security Mechanism Based on Hospital Authentication Server for Secure Application of Implantable Medical Devices

**DOI:** 10.1155/2014/543051

**Published:** 2014-07-24

**Authors:** Chang-Seop Park

**Affiliations:** Department of Computer Science, Dankook University, Cheonan 330-714, Republic of Korea

## Abstract

After two recent security attacks against implantable medical devices (IMDs) have been reported, the privacy and security risks of IMDs have been widely recognized in the medical device market and research community, since the malfunctioning of IMDs might endanger the patient's life. During the last few years, a lot of researches have been carried out to address the security-related issues of IMDs, including privacy, safety, and accessibility issues. A physician accesses IMD through an external device called a programmer, for diagnosis and treatment. Hence, cryptographic key management between IMD and programmer is important to enforce a strict access control. In this paper, a new security architecture for the security of IMDs is proposed, based on a 3-Tier security model, where the programmer interacts with a Hospital Authentication Server, to get permissions to access IMDs. The proposed security architecture greatly simplifies the key management between IMDs and programmers. Also proposed is a security mechanism to guarantee the authenticity of the patient data collected from IMD and the nonrepudiation of the physician's treatment based on it. The proposed architecture and mechanism are analyzed and compared with several previous works, in terms of security and performance.

## 1. Introduction

Implantable medical devices (IMDs), such as implantable cardiac defibrillators, insulin pumps, and neurostimuli, monitor chronic disorder within the body and perform life-critical functions to treat cardiac arrhythmia, diabetes, and Parkinson's disease. For instance, implantable cardioverter defibrillators sensing cardiac events can store measurements such as electrocardiograms and administer an electrical shock to restore a normal heart rhythm, when a rapid heartbeat is sensed. Healthcare practitioners can extract data from IMD or modify its settings wirelessly, using an external device called a “programmer.” Modern IMDs also support delivery of telemetry, for remote monitoring over long-range, high-bandwidth wireless links, so that chronic patients can be continuously monitored at home or work, without visiting a hospital [1, 2].

Recently, the privacy and security risks of IMDs have been widely recognized in the medical device market and research community [3], after two experimental security attacks have been reported, against a commercial implantable cardiac defibrillator and an insulin pump [4, 5]. As well as securing the wireless link between IMD and programmer, access to IMD should be carefully controlled to prevent illegal exposure of the patient data and life-threatening modification of its settings. Unfortunately, up-to-date commercial IMDs do not employ security mechanisms.

During the last few years, a lot of researches have been carried out addressing the privacy and security issues of IMD. Most of them have concentrated on the authenticated key establishment problems for setting up a secure channel between IMD and programmer. Even though security mechanisms [6] based on public-key cryptography have been proposed to establish a common secret between them, they are not suitable for resource-constraint IMDs, in terms of both computational complexity and energy consumption. Hence, the IMD security should either be based on lighter-weight symmetric encryption and authentication schemes [4, 7, 8] or employ a resource-rich personal device (e.g., smart phone) to mediate communication between an IMD and an external programmer [9–11]. Another approach for the IMD security is associated with the accessibility issues of IMDs, when an emergency situation occurs. Suppose an unconscious patient with IMD enters an emergency room (ER) of a non-primary-care hospital. In order for ER personnel to access the IMD the patient has, some backdoors should be integrated for the programmer. Even though several techniques [10–13] have been proposed, each of them has its own inherent security weaknesses.

In this paper, a new security architecture for the security of IMDs is proposed, based on a 3-Tier security model, where the programmer interacts with a Hospital Authentication Server (HAS) to get permissions to access IMDs. Regardless of the type of cryptography for the authenticated key establishment, it is assumed in the research carried out so far that there is a single programmer to take care of several IMDs and that the keying material of each IMD is stored in the programmer. However, there are many IMDs and programmers, where any IMD should be able to be accessed from any programmer. In this case, there are two problems, in terms of security and scalability. The keying material of each IMD should be preinstalled into each programmer, so that the scalability problem occurs. Furthermore, if one of the programmers falls into an adversary's hands and the keying materials of IMDs are exposed, the patient's privacy can be compromised by eavesdropping, and the patient's health might even be endangered, by allowing an unauthorized access to IMDs. To address these problems, HAS is introduced, which plays the role of distributing access keys to IMD, according to the physician's privilege. Instead of installing the keying materials of IMDs into several programmers, HAS both maintains the access keys to several IMDs and distributes them to each authorized physician, via the programmer.

A security mechanism is also proposed in this paper to guarantee the authenticity of the patient data collected from IMD and the nonrepudiation of the physician's treatment based on it. An authorized physician usually modifies IMD's settings according to the patient data read out from the patient's IMD, for the purpose of increasing the efficacy of the treatment. However, if the physician's treatment associated with IMD is not correct or appropriate, a medical accident might occur. It could be due to a medical malpractice or a medical error. In order to clearly resolve the medical dispute, a record of the physician's treatments based on the observed patient data will be valuable corroborated facts. Hence, the treatment record should be securely maintained, with nonrepudiation. Regarding this issue, another security mechanism is introduced to ensure that the patient data extracted from its IMD is not modified by the physician illegally, and a record of the physician's treatments based on the patient data cannot be modified, either, even by HAS.

After an introduction of the current commercial IMDs, together with their usage for pervasive patient monitoring in Section 2, related research works to improve the IMD security are presented in Section 3. Security requirements and models for IMDs are investigated in Section 4 to help understand the security mechanism proposed in Section 5. Finally, various features of the security mechanism proposed for the IMD security are analyzed in Section 6 and compared with other research works in terms of security and performance.

## 2. Current Commercial IMDs and Pervasive Monitoring Systems

IMDs are capable of measuring and altering the physiological characteristics of a patient. IMDs receive commands from an external device (programmer) that enable the adjustment of settings on the implanted device. They can also send information on their current status (as well as sensed data) to an external device. Modern IMDs contain the high-capacity lithium batteries that last five to seven years and the ultralow-power microprocessor with about 128 Kbytes of RAM for telemetry storage. Radio frequency (RF) telemetry, which operates within the Medical Implant Communications Service (MICS) Band, is used for wireless communication between IMD and programmer in the hospital or clinic. Using the MICS Band prevents interference with home electronics such as microwaves, cell phones, and baby monitors. The MICS Band (402 to 405 MHz) allows for 250 Kbps and read range of up to 5 meters.

**Figure 1:**
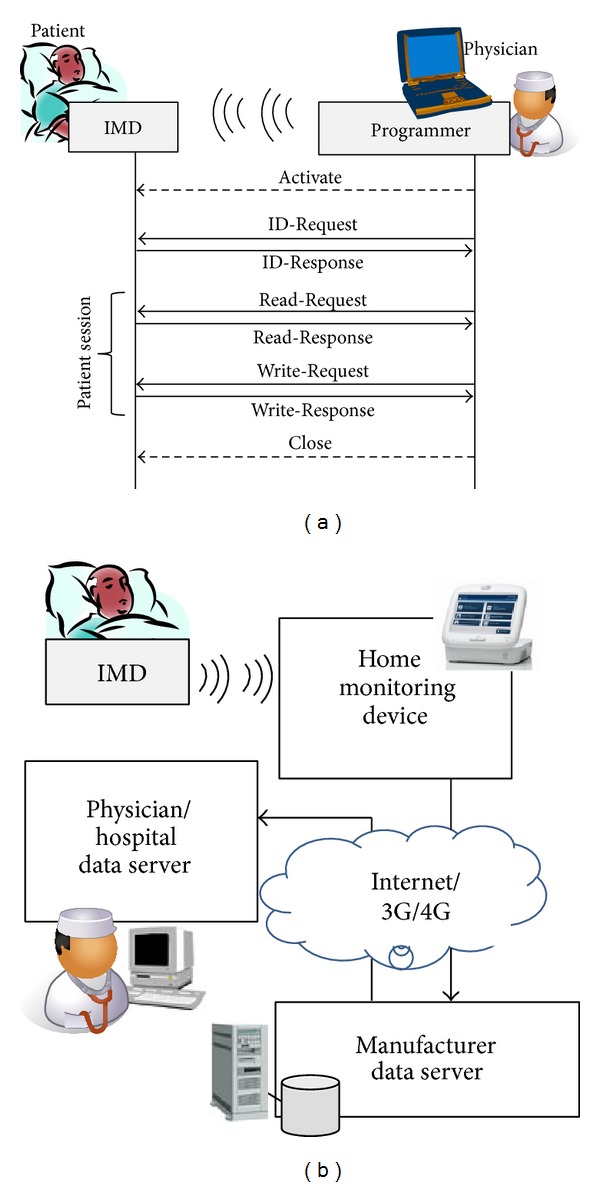
(a) Generic patient session protocol and (b) remote monitoring service for IMD patients.

To open a wireless communication channel between IMD and programmer, a magnet switch inside IMD should be activated by RF command. Then, the programmer searches for activated IMDs within telemetry range, by sending an* ID-Request* command. As shown in Figure 1(a), when receiving an* ID-Response* command from a target IMD, the programmer starts a patient session. The programmer interrogates IMD to gather measured data since the last patient session, through exchanging the* Read-Request* and* Read-Response* commands. IMD can also store patient-related information, such as patient name and date-of-birth, which can be viewed during a patient session. This information is typically programmed into IMD at the time of implant. The device settings for treatment can be reprogrammed into IMD, through the* Write-Request* and* Write-Response* commands. The* Close* command from the programmer ends the current patient session with IMD.

On the other hand, IMD manufacturers, such as Medtronics [14] and Biotronik [15], operate comprehensive Internet-based remote monitoring services to patients with their IMDs and their clinics. The home monitoring device is programmed to wake up IMD, per a schedule defined by physicians. Applicable data are then extracted from IMD and sent to the home monitoring device that communicates them to an Internet site that can be accessed by physicians as shown in Figure 1(b) [16]. Contrary to the programmer that can exchange both* Read* and* Write* commands, the home monitoring device can exchange the* Read* command only with IMD. Namely, the home monitoring device cannot be used to change the settings on IMDs for patient treatment. From now on, the security mechanisms applicable to Figure 1(a) are investigated.

## 3. Related Works

Since current commercial IMDs and programmers do not employ any security mechanisms for access control and transmission protection, a lot of research papers have been recently proposed to address the security issues associated with IMDs and programmers. Most of them are based on the secret information (e.g., password, secret key) preshared between IMDs and programmers, much like in other network security environments. Basically, the IMD security can be approached through access control at the programmer level. Only authorized personnel can access the programmer capable of communicating with IMD. However, if an adversary possesses his own programmer, this approach cannot be effective. The study in [7] proposed a password-based access control to IMD, where an adversary without knowledge of the password would be unable to access IMD. However, this does not provide any method to secure the transmission of the password to IMD. A challenge-response authentication has been proposed, based on the secret key shared between IMD and programmer [4, 8]. In particular, [4] is specific about the key management for the IMDs: the programmer keeps a master key *K*_*M*_, and each IMD has an IMD-specific key *K*_dID_ = *f*(*K*_*M*_, dID), where dID is a serial number or identity of IMD and *f*(·) is a pseudorandom function. So, given dID, the programmer can share *K*_dID_ with IMD, which can be used to gain access to IMD, either to send it commands or to read out medical data. Interestingly, a proximity-based access control scheme has been designed by [6]. Assuming that no secret information is shared between IMD and programmer, a session key is derived using a Diffie-Hellman key exchange. The authenticity of the Diffie-Hellman parameters exchanged is guaranteed by executing a distance bounding protocol [17, 18]: namely, wireless interaction with IMD is denied, unless the proximity of IMD is verified.

Personal devices, such as* Guardian* [9],* Communication Cloaker* [10], and* Shield* [11], have been introduced for the purpose of offloading long distance communication and intensive processing on the personal devices. In particular,* Guardian* plays the role of the key distribution center for IMD and programmer, assuming that* Guardian* has the public key of the programmer and a secret key is preshared between IMD and* Guardian*.

Some research efforts [10–13] on solving the tension between the security and the safety of IMD have been carried out, too. In emergency situations, where an unconscious patient with IMD enters an unfamiliar emergency room, the emergency medical staff would not have access to IMD, if a strict access control mechanism based on the preshared secret is employed, and the secret information is not available to them. In this situation, access to the IMD would be protected by a password that is engraved on the back of the medical bracelet the patient always wears or encoded as a 2D barcode and tattooed onto the patient's skin. In addition to regular tattoos with black or colored inks [12], it is now possible to get specialty tattoos that are only visible under UV lights [13].* Communication Cloaker* and* Shield* can be used for emergency situations. Namely, if they are removed from the patient, the system changes its access policy to allow any programmer to access IMD.

## 4. Security Requirements and Security Models for IMD

Since patient data should be available only to the authorized personnel, strict access control to IMD is indispensable, and the wireless link between IMD and programmer should be cryptographically protected. In particular, due to the wireless reprogramming capability, an adversary could conceivably cause direct physical harm to a patient, by sending commands to modify the treatment parameters of IMD. First, the wireless link should be protected, for both confidentiality and authenticity of the patient data. Second, the* Read-Request* and* Write-Request* commands in Figure 1 should be sent only by the authorized programmer (physician). Third, the authenticity of the patient data collected from IMD should be guaranteed, wherever it is stored. Fourth, the physician's treatment based on the collected patient data should be correct and careful to prevent medical accidents. Therefore, all the actions the physician takes for treatment should be recorded with nonrepudiation capability to solve the dispute when a medical accident occurs.

**Figure 2:**
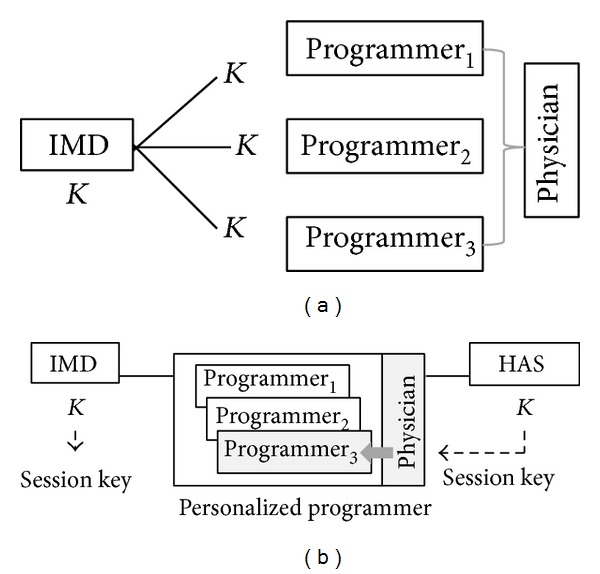
(a) 2-Tier security model and (b) 3-Tier security model.

Figure 2 shows two kinds of security models for IMD, the 2-Tier security model and 3-Tier security model. In order to use multiple programmers for a single IMD, a long-term key *K* should be stored into each programmer, as in Figure 2(a). Previous works for the IMD security are based on this 2-Tier security model. However, it is not easy to manage the long-term keys of IMDs in multiple programmers, when a lot of IMDs are present. In particular, if a programmer is stolen, the long-term keys of several IMDs can be exposed to the adversaries. In this paper, we propose a 3-Tier security model with the Hospital Authentication Server (HAS), as in Figure 2(b). There are multiple programmers handled by the physician. A basic function of each programmer is to send commands to IMD and to receive patient data from IMD. Even though each programmer does not store the long-term keys of IMDs, the common cryptographic modules (such as encryption and decryption algorithm) to secure the transmission between them are embedded into the programmer. On the other hand, HAS maintains the long-term keys of IMDs. The programmer is personalized, when the physician inserts his/her smartcard into it. The smartcard contains the physician's credentials, based on which session keys, derived from the long-term key, are passed securely to the personalized programmer. The session keys are eventually used to retrieve the patient data and to change the treatment parameters of IMD.

## 5. Proposed Security Mechanisms for the IMD

### 5.1. Assumptions and Design Principles

First, the security mechanism proposed here is based on the 3-Tier security model in Figure 2(b). There are many programmers available to physicians and patients. However, both patient- and physician-centric information, such as credentials, are not stored in the programmers. Second, the programmer is personalized, when the physician inserts his/her smartcard into it. The smartcard contains the long-term key *K*_pID_ preshared with HAS, as well as the physician's private key for signature SK_pID_. The physician's corresponding public key PK_pID_ for signature verification is stored in HAS. Third, a long-term key *K*_dID_ is preshared between IMD and HAS, when IMD is initialized for the first time. The IMD initialization process is shown in Section 5.2. Fourth, in order to access IMD, the physician should obtain access keys from HAS after successfully authenticating to HAS. There are two kinds of access keys: Read-Key and Write-Key. One is for retrieving the patient data from IMD, and the other is for granting a privilege to the physician to send a treatment command, such as device parameter changes. In particular, the Write-Key is generated by HAS based on the patient data retrieved from IMD, as well as corresponding treatment, so that the physician's treatment can be reactively verified, in the case of medical accident. Fifth, since HAS has all the security-related information of the patients, IMDs, and physicians, it is assumed that they are securely protected and maintained. The notations to be used in Sections 5 and 6 are summarized in Notations section.

### 5.2. IMD Initialization

**Figure 3:**
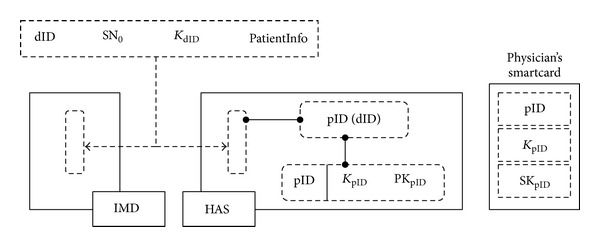
IMD initialization.

Prior to implanting IMD, it is initialized, by installing security-related information. As in Figure 3, the following security-related information {dID, SN_0_, *K*_dID_, PatientInfo} is securely stored into the IMD and HAS, where *K*_dID_ is the IMD's long-term key and SN_0_ is an initial patient session number.

During the IMD initialization phase, a list of primary care physicians, pID(dID), for the patient with IMD whose identification number is dID is determined. For each pID ∈ pID(dID), the corresponding physician's long-term key *K*_pID_ and public key PK_pID_ for signature verification are also stored in the HAS. The physician's smartcard contains pID, *K*_pID_, and private key SK_pID_ for signature generation.

### 5.3. Proposed Secure Patient Session

In order to initiate a new patient session through a programmer, the physician first personalizes the programmer, by inserting his/her smartcard containing both the long-term key *K*_pID_ and the physician's private key SK_pID_ into it. After exchanging the* ID-Request* and* ID-Response* commands during the IDENTIFICATION session, the personalized programmer obtains the device identification number dID of the target IMD, as well as the current session number SN_*j*_. To read from or write to IMD, the programmer requests permissions from HAS. The IDENTIFICATION session is performed based on the RFID to save the battery power on IMD. The current IMD is equipped with a RFID tag, and the programmer also has a RFID reader with it [19].

#### 5.3.1. READ Session

The READ session consisting of the four commands is for the personalized programmer, namely, physician, to read out the patient data stored in the target IMD. The* Read-Auth-Request* and* Read-Auth-Response* commands are protected by the session key derived from the physician's long-term key *K*_pID_ preshared with HAS, while both* Read-Request* and* Read-Response* commands are protected by the session key derived from the IMD's long-term key *K*_dID_, preshared with HAS.

(Step-①②) The personalized programmer first computes the session key *K*_*j*_ using the key derivation function *kdf*(·):

(1) $$\begin{array}{c} K_{j}=kdf\left(K_{\text{pID}},{\text{SN}}_{j},\text{dID},\text{pID}\right), \end{array}$$

where SN_*j*_ is the *j*th patient session number, dID is IMD's device identification number, and pID is physician's identification number. Then, the* Read-Auth-Request *{SN_*j*_, dID, pID, MAC(*K*_*j*_)} command is sent to HAS, where MAC(*K*_*j*_) is the message authentication code computed over all preceding fields of the command using *K*_*j*_. When receiving it, HAS also computes *K*_*j*_ and verifies MAC(*K*_*j*_). If the verification is successful, HAS computes the Read-Key, Kr_*j*_, based on *K*_dID_:

(2) $$\begin{array}{c} {\text{Kr}}_{j}=kdf\left(K_{\text{dID}},{\text{SN}}_{j},\text{dID},\text{pID}\right), \end{array}$$

which is sent back to the personalized programmer through the* Read-Auth-Response *{SN_*j*_, [Kr_*j*_]*K*_*j*_, MAC(*K*_*j*_)} command. The Read-Key is a kind of permission from HAS for the personalized programmer to access the patient data in IMD. Now, using the Read-Key, the* Read-Request* command is sent to IMD.

(Step-③④) When receiving the* Read-Request* command, IMD verifies the MAC value, after deriving the Read-Key, Kr_*j*_, in the same way as the HAS did. If the verification is successful, the PatientData_*j*_ and its integrity-check value Auth_*j*_ are sent to the personalized programmer, through the* Read-Response* command. Otherwise, IMD just drops the command and takes no further action. The integrity-check value Auth_*j*_ = *h*(*K*_dID_, SN_*j*_, PatientData_*j*_) guarantees that the PatientData_*j*_ is really sent from IMD. However, the personalized programmer cannot verify the integrity-check value, since it does not have the long-term key *K*_dID_. When receiving the* Read-Response* command, the programmer also checks the MAC value and obtains the PatientData_*j*_. If the purpose of initiating this patient session is only to retrieve the current patient data, the patient session is terminated here.

#### 5.3.2. WRITE Session

The WRITE session also consists of the four commands whose purpose is for the patient treatment, by adjusting the running parameters of IMD. In order for the personalized programmer to obtain permission for the write operation to IMD, the Write-Key is provided to the personalized programmer by HAS. In particular, since the physician's treatment, Conf_*j*_, should be based on the PatientData_*j*_ collected from IMD during the READ session, both PatientData_*j*_ and Conf_*j*_ should be applied, when deriving the Write-Key.

(Step-*❶* *❷*) If a subsequent action, such as changing parameters of IMD, should be taken for the treatment, based on the retrieved patient data, the personalized programmer should obtain a Write-Key from HAS. The personalized programmer generates the treatment command Conf_*j*_ based on PatientData_*j*_ and computes *Z*_*j*_ and Sig_*j*_, where

(3) Zj=[PatientDataj,Confj]Kj,Sigj=Sig(SKpID)  is obtained from  Wj,Wj=[SNj,dID,pID,PatientDataj,Confj,Authj,Sig(SKpID)].

*Z*_*j*_ is the encryption of the confidential data (PatientData_*j*_,Conf_*j*_) and Sig_*j*_ = Sig(SK_pID_) is the signature computed over [SN_*j*_, dID, pID, PatientData_*j*_, Conf_*j*_, Auth_*j*_] using the physician's private key SK_pID_. Since *W*_*j*_ contains the physician's treatment, Conf_*j*_, based on the patient physiological data, PatientData_*j*_, it can be used for evidence data when a medical dispute occurs. When receiving the* Write-Auth-Request *{SN_*j*_, dID, pID, *Z*_*j*_, Auth_*j*_, Sig_*j*_, MAC(*K*_*j*_)} command, HAS first verifies both the MAC value and the signature value and checks if the received Auth_*j*_ is identical to the computed Auth_*j*_. If the verifications are successful, HAS stores *W*_*j*_ in its database after obtaining Data_*j*_ and Conf_*j*_ from *Z*_*j*_. Finally, the Write-Key, Kw_*j*_, is computed and sent to the personalized programmer, through the* Write-Auth-Response *{SN_*j*_, [Kw_*j*_]*K*_*j*_, MAC(*K*_*j*_)} command:

(4) Kwj=kdf(KdID,SNj,dID,pID,PatientDataj,h(Configj)).

(Step-*❸* *❹*) After receiving the* Write-Request *{SN_*j*_, pID, [Conf_*j*_]Kw_*j*_, *h*(Conf_*j*_), MAC(Kw_*j*_)} command, IMD first computes its own Kw_*j*_ and verifies the MAC value. A reason to use *h*(Conf_*j*_) instead of Conf_*j*_ to generate the Write-Key is for confidentiality of the treatment parameters. If the verification is successful, IMD changes its parameters according to Conf_*j*_ and responds with the* Write-Response *{SN_*j*_, Status_*j*_, MAC(Kw_*j*_)} command, where Status_*j*_ is “*successful*.”

## 6. Analysis and Comparisons

### 6.1. Threat Model and Security Assumptions

We consider two types of adversaries with polynomially bounded computational power: an outside adversary and an inside adversary as in Figure 5. The outside adversary monitors and manipulates the wireless links among IMD, the personalized programmer, and HAS, for the purpose of accessing the patient data and modifying the physician's treatment to the patient. On the other hand, the inside adversary, including the physician, tries to falsify the patient data retrieved from IMD and to give unsuitable treatment to the patient. When a medical accident occurs, it might be due to unintentional medical malpractice or intentional error. Whether or not it is intentional, the effect of the medical malpractice and error is harmful to the patient. Therefore, all the actions the physician takes for treatment should be recorded with nonrepudiation capability to solve the dispute when a medical accident occurs. Denial-of-service attacks, such as an energy depletion attack, are excluded from our adversary model.

As mentioned in Section 5.1, it is assumed that two long-term keys, *K*_dID_ of IMD and *K*_pID_ of the physician, are preestablished. The physician's public key PK_pID_ is also enrolled in HAS, and the corresponding private key SK_pID_ is kept by the physician. It is assumed here that they are securely protected.

### 6.2. Formal Verification of the Proposed Protocol

**Figure 4:**
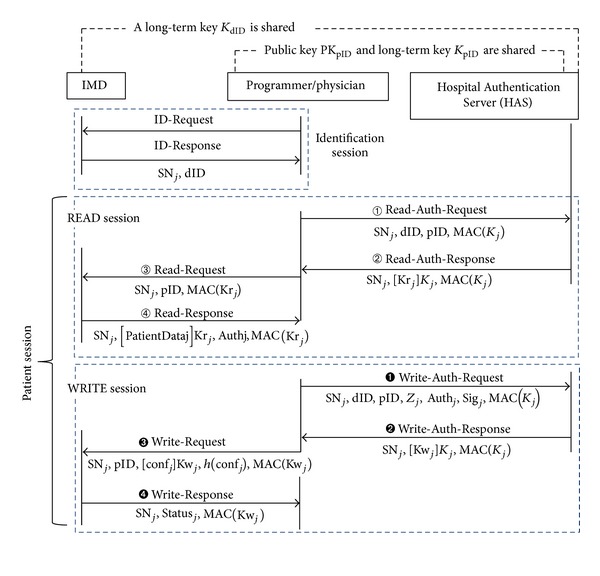
Proposed protocol for secure patient session.

**Figure 5:**
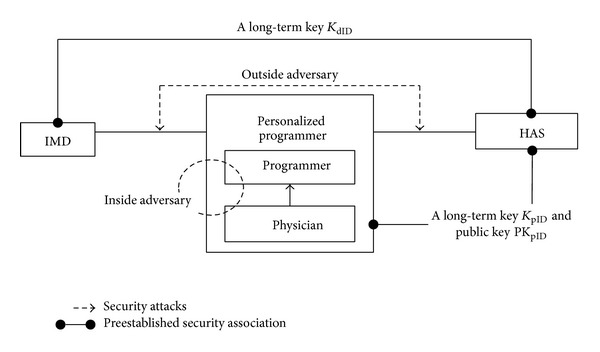
Threat model and preestablished security associations.

When designing a cryptographic protocol such as the one in Figure 4, one often uses arguments such as “since this message was digitally signed by one principal *A*, a second principal *B* can be sure it came from *A*” in informal proofs justifying how the cryptographic protocol works. However, in such informal proofs, it is easy to overlook an essential assumption, such as a trust relation or the belief that a message is not a replay from a previous session. Therefore, it is desirable to write such proofs in a formal method to help in pointing the weaknesses of the proposed cryptographic protocol.

There are three principals (programmer, IMD, and HAS) in the protocol proposed in Figure 4. What should be proven through the formal method is that the session key shared between any two principals is not exposed to the attackers (key authentication) and one principal is assured that a second principal actually has possession of the session key (key confirmation).

**Figure 6:**
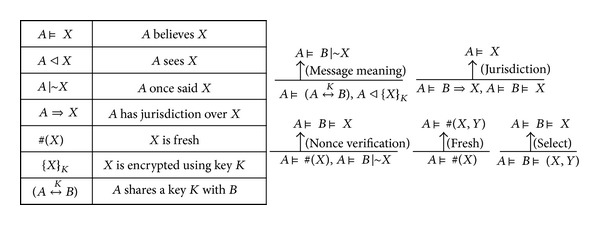
Notations and inference rules of BAN logic.

In this section, the correctness of the proposed cryptographic protocol is formally validated, based on BAN logic. The notations and five inference rules of the BAN logic are shown in Figure 6. The symbols *A* and *B* denote specific principals involved in a particular protocol, while *X* and *Y* are formulas. The details on notations and logical postulates of the BAN logic can be found in [20]. In particular, the definition of {*X*}_*K*_ is extended to denote both encryption of *X* with *K* and that*X* is integrity protected with *K*, since the purpose of the rule $\left\{A⊨B|\sim X\;\text{derived}\;\text{from}\;A⊨(A\overset{k}{↔}b\right)$,  *A*⊲{*X*}_*K*_} is to verify that *X* is sent from *B*.

We verify the correctness of the proposed protocols, the READ session and WRITE session, of Figure 4. However, since the two protocols are identical in terms of the way of deriving and using the session keys, the correctness of the READ session protocol is verified. For simplicity of notations, *P*, *H*, and *I* denote programmer/physician, HAS, and IMD, respectively. The idealized “READ session” protocol of Figure 4 is formulated as follows:

(5) $$\begin{array}{c} P⟶H:{\left\{{\text{SN}}_{j},P\overset{K_{j}}{↔}H\right\}}_{K_{j}},\\ P⟵H:{\left\{{\text{SN}}_{j},{\left\{P\overset{{\text{Kr}}_{j}}{↔}I\right\}}_{K_{j}}\right\}}_{K_{j}},\\ I⟵P:{\left\{{\text{SN}}_{j},{\left\{P\overset{{\text{Kr}}_{j}}{↔}I\right\}}_{{\text{Kr}}_{j}}\right\}}_{{\text{Kr}}_{j}},\\ I⟶P:{\left\{{\text{SN}}_{j},{\left\{P\overset{{\text{Kr}}_{j}}{↔}I\right\}}_{{\text{Kr}}_{j}}\right\}}_{{\text{Kr}}_{j}}. \end{array}$$

The following are the initial assumptions for analysis, while some assumptions unused for analysis are omitted here:

(6)P⊨H⟹(P↔KrjI),I⊨(P↔KrjI),P⊨#(SNj),I⊨#(SNj),(7)P⊨(P↔KjH)/∗since  Kj  is  derived  from  KpID  shared between  P  and  H∗/,(8)H⊨(P↔KjH)/∗since  Kj  is  derived  from  KpID  shared between  P  and  H∗/.

**Figure 7:**
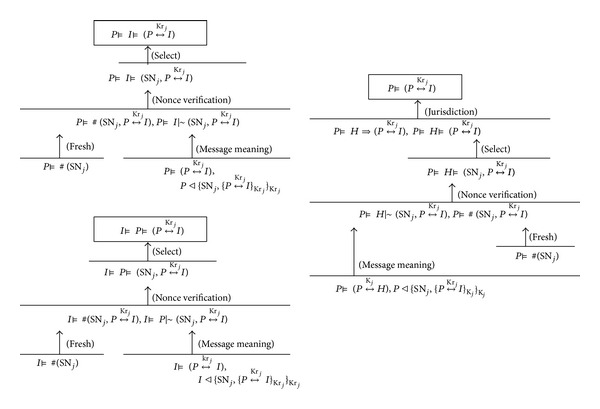
Formal verification for correctness of the proposed protocol.

The final goal of the protocol is $P\;\;⊨\;\;(P\overset{{\text{Kr}}_{j}}{↔}I)$, $I⊨(P\overset{{\text{Kr}}_{j}}{↔}I)$, $P\;\;⊨\;\;I\;\;⊨\;\;(P\overset{{\text{Kr}}_{j}}{↔}I)$, and $I\;\;⊨\;\;P\;\;⊨\;\;(P\overset{{\text{Kr}}_{j}}{↔}I)$, where $I\;\;⊨\;\;(P\overset{{\text{Kr}}_{j}}{↔}I)$ is an initial assumption, and no proof is needed. The first two goals guarantee the mutually authenticated key establishment (*Key Authentication*) between *P* and *I*, while the other two goals are for* Key Confirmation*, which is the property whereby one principal is assured that a second principal actually has possession of the key Kr_*j*_. As shown in Figure 7, both* Key Authentication* and* Key Confirmation* properties are embedded into the READ session protocol of Figure 4.

### 6.3. Forgery and Replay Attacks by an Outside Adversary

All the commands between IMD and personalized programmer are secured using the Read-Key, Kr_*j*_ = *kdf*(*K*_dID_, SN_*j*_, dID, pID), and Write-Key, Kw_*j*_ = *kdf*(*K*_dID_, SN_*j*_, dID,pID, PatientData_*j*_, *h*(Config_*j*_)), which are a kind of session keys for the current patient session. Since IMD/physician identification numbers, dID and pID, as well as the current session number, SN_*j*_, are used to derive the session keys, the freshness of each command can be guaranteed to protect from a replay attack. In particular, the patient data PatientData_*j*_ and physician's treatment Conf_*j*_ are symmetrically protected against eavesdropping, for patient privacy. On the other hand, the session key, *K*_*j*_ = *kdf*(*K*_pID_, SN_*j*_, dID, pID), derived from the physician's long-term key, *K*_pID_, is employed to secure commands exchanged between the HAS and personalized programmer. Hence, without knowing *K*_dID_ and *K*_pID_, it is not feasible to forge or replay the commands.

### 6.4. Mutual Authentication between Physician and HAS

The* Read-Auth-Request* command (①) plays the roles of both authenticating the personalized programmer (namely, physician) to the HAS and sharing the session key, *K*_*j*_ = *kdf*(K_pID_, SN_*j*_, dID, pID), with HAS. The physician authentication is based on his/her long-term key, K_pID_, shared with the HAS:

(9) $$\begin{array}{c} Read\text{-}Auth\text{-}Request\left\{{\text{SN}}_{j},\text{dID},\text{pID},\text{MAC}\left(K_{j}\right)\right\},\\ Read\text{-}Auth\text{-}Response\left\{{\text{SN}}_{j},\left[{\text{Kr}}_{j}\right]K_{j},\text{MAC}\left(K_{j}\right)\right\}. \end{array}$$

The HAS maintains a list of primary care physicians PID(dID) authorized to access each dID registered. If pID ∈ PID(dID), then a Read-Key, Kr_*j*_, is granted to the physician through the* Read-Auth-Response* command (②). Otherwise, the command is silently dropped, or an error message is sent back to the physician. In particular, the successful verification on the MAC value of the* Read-Auth-Response* command corresponds to the successful authentication on the HAS.

### 6.5. Protection against Patient Data Falsification

A reason to include pID in the* Read-Request* command is for IMD to compute a session key, Kr_*j*_ = *kdf*(*K*_dID_, SN_*j*_, dID, pID), to be shared with the personalized programmer. The* Read-Response* command contains two fields: [PatientData_*j*_]Kr_*j*_ and Auth_*j*_, where PatientData_*j*_ is encrypted for the patient privacy and Auth_*j*_ is the origin-check value for PatientData_*j*_:

(10) Read-Request{SNj,pID,MAC(Krj)},Read-Response{SNj,[PatientDataj]Krj,Authj,MAC(Krj)}.

The current programmer stores the patient data in plaintext form retrieved from IMD into its internal or external memory. Since the patient data is not protected at all, as long as it remains in the programmer, there is a possibility that it can be modified intentionally or accidentally, before it is sent to HAS. Even though the physician is authorized to retrieve the patient data, its origin and integrity should be guaranteed, so that even the physician cannot modify it. Hence, the patient data is accompanied by the origin-check value Auth_*j*_ = *h*(*K*_dID_, SN_*j*_, PatientData_*j*_). Since the origin-check value is computed based on the IMD's long-term key, *K*_dID_, shared between IMD and HAS, it can be verified by HAS when it is finally stored in the HAS.

### 6.6. Authenticity of the Physician's Treatment with Nonrepudiation

After examining the patient data, PatientData_*j*_, retrieved from IMD, the physician generates a treatment, Conf_*j*_, based on which IMD's running parameters are changed. However, if the physician's treatment associated with IMD is not correct or appropriate, a medical accident might occur. In order to clearly resolve the medical dispute, a record of the physician's treatments based on the observed patient data will be valuable corroborated facts. Hence, the treatment record should be securely maintained with nonrepudiation. In order for the physician to obtain the Write-Key, Kw_*j*_, from HAS, he/she must digitally sign both patient data and the corresponding treatment as follows:

(11) Write-Auth-Request{SNj,dID,pID,Zj,Authj,Sigj,MAC(Kj)}, whereSigj=Sig(SKpID)is  obtained  from  Wj,Wj=[SNj,dID,pID,PatientDataj,Confj,Authj,Sig(SKpID)],Write-Auth-Response{SNj,[Kwj]Kj,MAC(Kj)}, whereKwj=kdf(KdID,SNj,dID,pID,PatientDataj,h(Configj)).

Suppose the physician is an inside adversary. Then, the following two security attacks can be imagined: the first is to generate an abnormal treatment, given the correct patient data. The second is to modify the patient data and generate a normal treatment corresponding to the modified patient data. In the first case, if a medical accident occurs, the liability of the physician is proven, due to the physician's signature. In the second case, it is not possible to modify the patient data arbitrarily by the physician, since it is protected with Auth_*j*_ = *h*(*K*_dID_, SN_*j*_, PatientData_*j*_), as described in Section 6.5.

### 6.7. Emergency Care Issues

In emergency situations, where an unconscious patient with IMD should be taken care of in either an unfamiliar emergency room of a different hospital or an emergency vehicle, IMD should first be disabled for emergency care. However, the emergency medical staff would not have access to IMD, if the access key is not available. Several methods [10–13], such as wearing personal devices or medical bracelets with the password/secret key, have been proposed to access IMD under such emergency situations. They can be effective methods, as long as the personal device or medical bracelet is not lost. Here, the medical bracelet method is employed for emergency care:

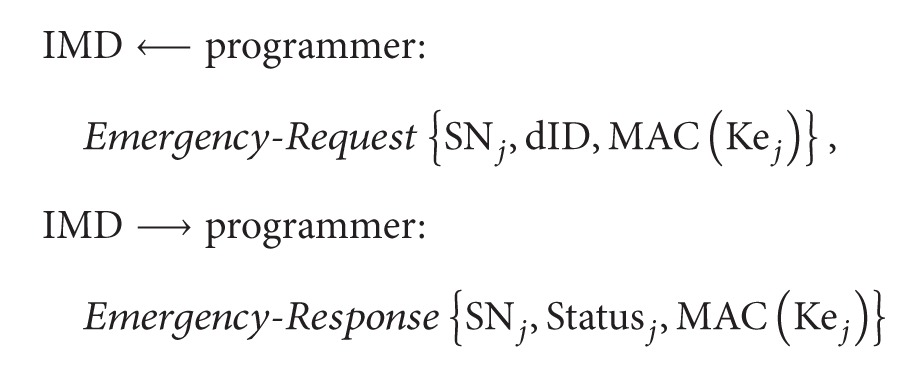
(12)
Suppose a patient wears a medical bracelet with *K*_dID_. Then, the emergency key, Ke_*j*_ = *kdf*(*K*_dID_, SN_*j*_, dID), is derived by the programmer, after obtaining both SN_*j*_ and dID during the IDENTIFICATION session, and the* Emergency-Request* command is sent to IMD to disable it.

### 6.8. Security Comparisons

In this section, various security features of the proposed protocol are compared with those in [4, 6, 8, 9]. Security comparisons are summarized in Table 1. The proposed one and the one in [9] are based on the 3-Tier security model. However, [9] employs a personal device with more power and computational resources than IMD. It works as a proxy for IMD and performs the authentication on its behalf.

Except [8], cryptographic methods for the authentication of IMD and programmer are challenge-response authentication based on a shared secret. In particular, the challenge-response authentication in [9] is based on a shared secret between IMD and personal device and digital signature between the personal device and programmer. The proposed one and those in [6, 9] generate session keys for confidentiality, integrity, and authentication. The session key between IMD and programmer is generated based on the Diffie-Hellman key exchange in [6], by the personal device in [9], and by HAS in the proposed one.

The key management scheme is employed in the proposed one and in [4, 9]. In [9], the personal device maintains the public keys of multiple programmers. The public key is used for the personal device to send the session key to the programmer and to verify the signature generated by the programmer. The IMD-specific keys, *K*_dID_ = *f*(*K*_*M*_, dID), are derived from a master key, *K*_*M*_, which is common to all the programmers in [4]. When receiving the IMD's identification number, dID, from the IMD, the programmer can share the IMD-specific key with IMD.

In the case that a programmer is stolen by an adversary, the security of IMD can be endangered. Since the master key and private key of the programmer are stored in the programmer in [4, 9], respectively; they are insecure. On the other hand, [6] employs the Diffie-Hellman key exchange to generate the session key, so that the stolen programmer does not induce the security problem of IMD. Likewise, the proposed one is also secure, since secret information is not stored in the programmer. The last two features, Patient Data Falsification and Medical Dispute Resolution, are provided only by the proposed one, which has been discussed in Sections 6.5 and 6.6, respectively.

The proposed protocol in Figure 4 has a similarity to the Kerberos protocol [21] in terms that session keys are shared between IMD and programmer through HAS. However, there are several differences between them, so that the Kerberos protocol cannot be applied directly to the proposed protocol. First, unlike the Kerberos protocol that is distributing only one session key, two distinct session keys (Read-Key, Write-Key) are employed in the proposed protocol in order to differentiate between* Read* permission and* Write* permission. Second, the session key is encrypted and transported to IMD from HAS when employing the Kerberos protocol, while only the keying materials to compute the session key are transported to IMD in the proposed protocol. Third, to prevent from the replay attack, the timestamps are used in the Kerberos protocol, while the session numbers are employed in the proposed protocol. Basically, the timestamp for freshness is not appropriate for the IMD-programmer environment since timestamp-based protocols require that time clocks be both synchronized and secured. Namely, a supplementary protocol for the time synchronization between IMD and programmer should be provided. Furthermore, it is not difficult to modify the local time clock of the programmers.

### 6.9. Performance Comparisons

Since each of [4, 6, 8, 9] is devoted to its own security feature that is not adequate for direct comparison, the proposed one and those in [6, 9] are compared in terms of computational complexity of the authentication/integrity, session key generation/distribution, and the number of messages exchanged. Performance comparisons are summarized in Table 2.

In order to generate a session key, *g* ^*xy*^ mod *p*, between IMD and programmer, [6] uses a DH (Diffie-Hellman) key exchange, where *g* ^*x*^ mod *p* and *g* ^*y*^ mod *p* are DH contributions generated by each of them and exchanged via rapid bit exchange. The rapid bit exchange (RBE) means that each bit of the DH contributions is transmitted on a bit-by-bit basis to estimate the distance between them. So, a total of 4 · |*p*| messages, each of which consists of one bit, are exchanged, where 2 · |*p*| are for the bit-by-bit exchanges of two additional nonces. Then, the session key is used for the programmer to authenticate itself to the IMD. Only one MAC (message authentication code) computation is required for unilateral authentication.

The proposed one and that in [9] are based on the 3-Tier security model with PD (personal device) and HAS (Hospital Authentication Server), respectively. In the case of [9], PD generates and encrypts a session key with the public key of the programmer and the shared key with IMD, respectively, and sends it to them. The session key is used to protect the command and response exchanged between IMD and the programmer. The programmer is unilaterally authenticated to PD, based on the signature, while IMD unilaterally authenticates PD, based on the preshared secret. A total of 6 messages are exchanged among IMD, PD, and programmer.

In the proposed one, there are two distinct sessions, the READ and WRITE sessions, for a patient session. Since they are identical in terms of computational complexity, only the READ session is considered for equivalent comparisons with [6, 9]. The (personalized) programmer exchanges two messages with HAS for the purpose of obtaining the Read-Key and exchanges another two messages with IMD to obtain the current patient data. A total of 4 messages are exchanged among IMD, programmer, and HAS. On the other hand, during the WRITE session, the signature is used for both the patient data protection and medical dispute resolution, which are not supported in [6, 9].

## 7. Conclusions

Recently, the privacy and security issues of IMDs have been widely recognized in the medical device market and research community, and a lot of researches have been carried out to address the privacy and security issues of IMDs. Most of them have concentrated on the authenticated key establishment between the IMD and programmer, as well as solving the tension between the security and the safety of IMDs in emergency situations. By introducing a new security architecture in this paper, we have addressed three security-related issues associated with the IMDs: support for multiple programmers, prevention of patient data falsification, and the physician's treatment protection. In particular, by introducing HAS, multiple programmers can be supported for the IMDs, without embedding the keying materials inside the programmers. Furthermore, even the physician cannot modify the patient data arbitrarily, and the physician's treatment history based on the observed patient data can be monitored securely.

## Figures and Tables

**Table 1:** Security comparisons.

	[8]	[4]	[6]	[9]	Proposed
Security model	2-Tier	2-Tier	2-Tier	3-Tier w/PD	3-Tier w/HAS
Cryptographic method for authentication	Password	CR with shared secret	CR with shared secret	CR with shared secret and signature	CR with shared secret
Authentication type	Unilateral	Unilateral	Mutual	Unilateral	Mutual
SK generation	None	None	Diffie- Hellman	PD generates SK	HAS generates SK
Key management	N/A	IMD-specific key from MK	N/A	PD maintains multiple PKs	RK and WK from LK
Multiple programmers	N/A	Insecure support	Secure support	Insecure support	Secure support
Patient data protection	N/A	N/A	N/A	N/A	Secure with Auth_*j*_
Treatment protection	N/A	N/A	N/A	N/A	Possible with Auth_*j*_ and Sig_*j*_

CR: challenge-response; PD: personal device; MK: master key; SK: session key; LK: long-term key; PK: public key; RK: Read-Key; WK: Write-Key.

**Table 2:** Performance comparisons.

2009	[6]		[9] w/PD			Proposed w/HAS		
	IMD	Prog.	IMD	PD	Prog.	IMD	Prog.	HAS
Authentication/integrity	1 mac (UniL)	1 mac (UniL)	1 enc 2 dec	1 enc 1 ver	2 enc/1 dec 1 sig	2 mac	4 mac	2 mac
SK generation/distribution	1 DH w/RBE	1 DH w/RBE	1 dec	1 Penc 1 enc	1 Pdec	1 kdf	1 dec	1 kdf 1 enc
No. msg	4 *·*|*p*|		IMP-PD: 1/PD-Prog.: 3 IMD-Prog.: 2			IMD-Prog.: 2/Prog.-HAS: 2		
Patient data protection	N/A		N/A			1 enc 1 hash	1 enc 1 dec	1 hash 1 dec
Treatment protection	N/A		N/A			N/A	1 sig	1 ver

PD: personal device; Prog.: programmer; No. msg: number of messages exchanged; mac: MAC function; UniL: unilateral authentication; RBE: rapid bit exchange; DH: Diffie-Hellman; hash: hash function; Penc: public-key encryption; Pdec: public-key decryption; enc: symmetric encryption; dec: symmetric decryption; kdf: key derivation function; sig: signature generation; ver: signature verification.

## Notations

dID: IMD's device identification number
pID: Physician's identification number
PatientInfo: Patient information such as patient name, date-of-birth, and gender
SN_*j*_: The *j*th patient session number
PatientData_*j*_: Patient data collected from IMD during the *j*th patient session
Conf_*j*_: Physician's treatment to change parameters of IMD during the *j*th patient session
MAC(*K*): Message authentication code computed over all preceding fields of a command using symmetric key *K*; namely,{*m*, MAC(*K*) = *h*(*K*, *m*)}, where *m* is the command and *h*(·) is one-way hash function
[*m*]*K*: Encryption of *m* using symmetric key *K*
*kd**f*(·): Key derivation function
*h*(·): One-way hash function
*K*_dID_: A long-term key shared between IMD and HAS
PK_pID_, SK_pID_: Physician's public key and private key for signature
*K*_pID_: A long-term key shared between physician and HAS
Kr_*j*_, Kw_*j*_: Read-Key and Write-Key for the *j*th patient session
*K*_*j*_: Session key derived from *K*_pID_ for the *j*th patient session
Sig(SK_pID_): Signature computed over all preceding fields of a command using the physician's private key.
